# Case Report: Endoscopic submucosal tunneling dissection using a golden knife for a giant gastric cancer lesion: a case report and literature review

**DOI:** 10.3389/fonc.2025.1450446

**Published:** 2025-05-30

**Authors:** Pan Wu, Xi-Feng Jin

**Affiliations:** ^1^ Nursing Department, The Second Affiliated Hospital, Zhejiang University School of Medicine, Zhejiang University, Hangzhou, China; ^2^ Departments of Gastroenterology, The Second Affiliated Hospital, Zhejiang University School of Medicine, Zhejiang University, Hangzhou, China

**Keywords:** ESTD, ESD, golden knife, gastric cancer, giant gastric cancer lesion

## Abstract

**Background:**

Endoscopic Submucosal Dissection (ESD) is a well-established technique for the removal of early gastrointestinal cancers. However, it is often perceived as time-consuming and carries a higher risk, particularly when dealing with larger lesions, especially those exceeding 3 cm in diameter. In this case report, we introduce the application of Endoscopic Submucosal Tunneling Dissection (ESTD) for the management of a substantial gastric superficial neoplasia, which encompassed a considerable area of early gastric cancer. Although there are several case reports detailing the use of ESTD for the resection of gastrointestinal cancers, there have been no documented instances of utilizing a “Golden Knife” specifically for the treatment of large gastric cancer lesions.

**Case presentation:**

This case report details the treatment of a 64-year-old male diagnosed with a large early-stage gastric cancer, measuring approximately 140 mm by 88 mm. The medical team opted for endoscopic submucosal tunneling dissection (ESTD) using a golden knife, a technique chosen for its effectiveness in managing such tumors. Following the procedure, pathological examination indicated a pT1a tumor of the tub2 type, with dimensions of 120 mm by 42 mm. Importantly, all assessments, including ulcer (UL), lymphatic (LY, vascular (V), histological margin (HM), and vascular margin (VM), returned negative results, suggesting no further spread of the cancer. However, post-surgery, the patient experienced gastric stenosis, necessitating additional interventions, which included the placement of a nutritional tube and dilation of the stenosis to alleviate symptoms. The intraoperative strategies employed during the ESTD procedure, along with coordinated care and psychological support throughout the recovery process, played a crucial role in helping the patient regain confidence. This comprehensive approach ultimately contributed to satisfactory outcomes in his recovery journey.

**Conclusion:**

In conclusion, ESTD offers a safer and more effective alternative to traditional methods in specific cases, especially for patients with large or challenging gastric lesions who favor a minimally invasive approach.

## Introduction

Endoscopic submucosal dissection (ESD) is a well-established technique for the resection of early gastrointestinal cancers, but it relies heavily on maintaining a clear operative view to ensure successful outcomes. This can be particularly challenging when dealing with large lesions, especially those exceeding 3 cm in diameter, as conventional ESD is often perceived as both time-consuming and high-risk in such cases. The procedure typically involves making a circumferential incision to delineate the boundaries of the lesion. However, this method can inadvertently cause the injected fluid to diffuse, which shortens the duration of the submucosal fluid cushion that is critical for visibility and stability during the procedure. Furthermore, as the resected mucosa shrinks, the clarity of the submucosal endoscopic view may diminish, complicating the dissection process and potentially impacting the overall effectiveness of the treatment (1).

Performing operations on the lesser gastric curvature, particularly at the gastric angle, presents unique challenges that can complicate the procedure. These challenges stem from two main factors: first, the natural curves of the stomach and the thinner muscular layer in this area increase the risk of perforation; second, using the reverse gastroscope method makes it difficult to control the angle and direction of the cut. As a result, ESD for large lesions in the lesser curvature requires more time and demands highly skilled endoscopists to navigate these complexities. Although various endoscopic assist devices have been developed to address these issues, their effectiveness is still limited, which hinders their suitability for widespread standardized use in clinical practice (2–4).

To address these issues, ESTD has emerged as a novel approach for the effective and clear removal of circumferential superficial early tumors. In 2009, Linghu et al. successfully demonstrated the use of ESTD to excise circumferential superficial early-stage cancers of the stomach (SESCNs), marking the introduction of a new treatment strategy that improves both operational efficiency and visibility. Today, ESTD is utilized in clinical practice for treating not only SESCNs but also submucosal tumors (SMTs) (5–9). This study aims to clarify the outcomes of ESTD specifically for large gastric superficial neoplasms using a golden knife technique and to share our experiences in order to encourage its broader implementation in clinical settings.

## Case summary

In March 2023, a 64-year-old male was admitted to the hospital with suspected gastric carcinoma. Gastroendoscopy and CT scans identified lesions located in the gastric antrum, angularis, and lower corpus. Further evaluation using Narrow Band Imaging and Magnifying Endoscopy (NBI with ME) revealed that the lesions had a brownish appearance, indicating a potential pathological condition.Additionally, irregular microsurface patterns (IMSP+) and demarcation lines (DL+) were observed in the patient. Consequently, ESTD was chosen for this patient because of a large and difficult-to-access gastric lesion, which made traditional endoscopic resection methods unsuitable. Furthermore, the patient’s medical history excluded the possibility of surgical intervention, positioning ESTD as a safer and less invasive option. The procedure was carried out by an experienced endoscopist with over nine years in the field of endoscopic submucosal dissection, whose skills are supported by published research (10, 11).

**Figure 1:**
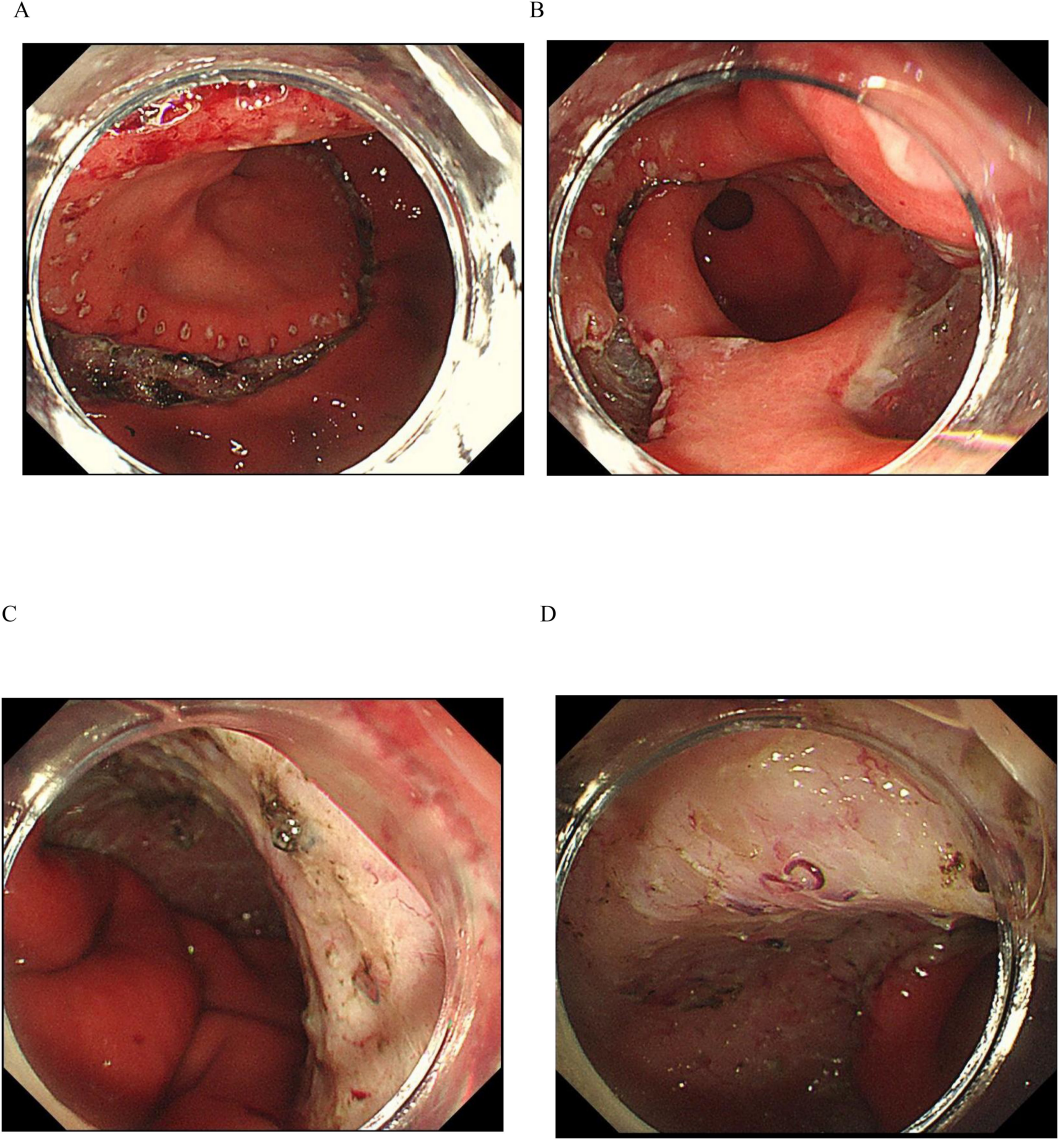
ESTD Procedure. **(A)** The margin was demarcated using an electrosurgical knife. **(B)** Sequential incisions were made at the anal and oral ends with a gold knife to create a submucosal tunnel. **(C, D)** The endoscopic view shows the lesion after the ESTD procedure.

For the ESTD surgery, the team utilized the Nanjing Weichuang Medical golden knife (model MK-T-1-195), a device approved for clinical use by the National Medical Products Administration (NMPA) of China, as noted by Certification No. 2018322025. This innovative surgical instrument integrates submucosal injection, electrocoagulation, and cutting functions, which streamlines the procedure by eliminating the need for multiple needle exchanges and various mucosal cutting tools, thereby saving significant time. After performing a circumferential mucosal incision, we employed dental floss traction and tunneling dissection techniques to improve visibility, delineate the submucosal layer, and ensure the complete excision of the lesion (refer to **Figures 1A-D** and **Supplementary Figures 1A, B**). The excised specimen measured approximately 140 mm by 88 mm.

The macroscopic type of the lesion was classified as type 0-IIb+IIa+IIc. A total of 39 slides were prepared for analysis. Histological examination revealed that the lesion was a moderately differentiated adenocarcinoma, measuring 120 mm by 42 mm. The tumor exhibited invasion into the submucosal layer and demonstrated focal involvement of the muscularis mucosa. Importantly, there was no evidence of vascular invasion, and both the horizontal and vertical margins were negative, indicating clear margins.

Post-operative assessments were scheduled at one month, three months, and six months, with additional evaluations occurring annually thereafter. The follow-up process included both clinical evaluations and imaging studies. The patient returned one month after surgery, reporting symptoms of acid regurgitation due to post-stenosis in the distal stomach and antrum. To address this, endoscopic balloon dilation was successfully performed at one, three, and six months, achieving a diameter of 20 mm (see **Supplementary Figures 2**-**4**). Notably, there was no recurrence of symptoms during the two-year follow-up period.

## Discussion

In this case report, we describe the treatment of a giant early gastric cancer utilizing Endoscopic Submucosal Tunneling Dissection (ESTD) in conjunction with the golden knife technique. Our findings add to the expanding evidence that highlights the safety and effectiveness of ESTD as a minimally invasive option compared to conventional surgical methods for addressing large gastric cancers.

ESTD represents a novel approach to the minimally invasive treatment of gastrointestinal tumors, especially those situated in difficult anatomical regions. The introduction of ESTD has created new opportunities for therapeutic interventions, offering alternatives to conventional techniques such as Endoscopic Submucosal Dissection (ESD) and Submucosal Tunnel Endoscopic Resection (STER). This technique is particularly beneficial for larger superficial esophageal neoplasms, whereas STER is mainly used for subepithelial tumors. When comparing ESTD with STER and ESD, there are notable differences in their technical methods, indications, and outcomes. Recent studies indicate that ESTD presents several advantages over traditional approaches, including enhanced visualization and shorter operation times, which could result in improved patient outcomes and reduced complication rates (12–14).

Recent studies have highlighted the operational challenges and learning curve associated with ESTD in comparison to ESD and STER. It has been observed that ESTD is generally less technically demanding than ESD, largely due to its more straightforward approach to submucosal dissection. In ESTD, the formation of a submucosal tunnel enhances the visibility of the dissection plane, which helps to lower the risk of complications that can arise from the obscured views often encountered in ESD procedures (3). Additionally, research suggests that the learning curve for ESTD is shorter, with many practitioners reaching a level of proficiency after fewer cases compared to what is typically required for ESD. This aspect is especially advantageous for less experienced endoscopists, as it facilitates a quicker adaptation to the technique and reduces the likelihood of complications during their initial learning phase (15, 16).

When comparing the treatment efficacy and tumor resection rates of ESTD to ESD, evidence indicates that ESTD may yield better outcomes. Meta-analyses have shown that ESTD achieves higher rates of en bloc resection and R0 resection compared to ESD, suggesting a more thorough removal of tumors and lower chances of residual disease (15, 17). Furthermore, the operational time for ESTD is typically shorter, which can enhance patient throughput and decrease the duration of anesthesia exposure. For example, one study found that the average resection speed for ESTD was 19.3 mm²/min, while for ESD it was 17.7 mm²/min, underscoring the efficiency of the ESTD technique (3, 6).

Complication rates are a pivotal factor in comparing ESTD with alternative techniques. Research indicates that ESTD is associated with significantly fewer complications than ESD. For example, one study found that the perforation rate in patients undergoing ESTD was only 0.9%, compared to 6.0% in those receiving ESD, which underscores the superior safety profile of ESTD (3, 13). Additionally, the overall incidence of adverse events, such as bleeding and infections, was lower in patients treated with ESTD. This reduction in complications can be attributed to the improved visualization and controlled dissection afforded by the submucosal tunnel technique, making ESTD a more favorable option for treating superficial gastrointestinal tumors (15, 17).

A significant concern associated with ESD, including ESTD, is the potential for post-surgery stenosis. This risk is particularly pronounced when the mucosal defect is extensive, such as when a lesion is resected circumferentially by more than three-quarters or when it exceeds 5 cm in length. Post-ESTD stenosis is most commonly seen in resections near the pylorus or cardia, where changes in anatomy can lead to gastric outlet obstruction. Although balloon dilation has proven effective in managing post-surgery stenosis, it often requires multiple procedures to fully restore the lumen’s patency and carries inherent risks, including the possibility of perforation. This highlights the importance of carefully selecting suitable candidates for ESTD and employing meticulous surgical techniques to minimize the risk of stenosis. Additionally, regular follow-up evaluations are essential for detecting early signs of stenosis, allowing for timely interventions and reducing the need for repeated dilation procedures.

A significant benefit of ESTD in this case is the use of the golden knife, a versatile tool that combines submucosal injection, electrocoagulation, and cutting functions into one cohesive system. This integration reduces the need for frequent instrument changes, which not only shortens the overall procedure time but also enhances operational continuity. The golden knife’s multifunctionality streamlines the process, making it particularly advantageous for larger lesions that would typically necessitate multiple instrument swaps during traditional ESD. Furthermore, minimizing instrument exchanges not only boosts the precision of the dissection but also lowers the risks of thermal injury and tissue damage, contributing to a safer and more effective procedure. To date, our team has employed this device in multiple cases involving large gastric lesions, resulting in positive outcomes and minimal complications. The device’s accuracy and intuitive design have greatly improved the safety and efficacy of ESTD.

The use of advanced tools in ESD, particularly the dental floss technique, has been shown in previous studies to enhance the precision of tissue dissection (18). The integration of ESTD with dental floss traction in this case significantly enhances the effectiveness of the procedure. In traditional ESD, once the circumferential incision is made, the lesion tends to retract, which can hinder visibility and stability in the surgical field, particularly with larger lesions. However, By using dental floss traction to secure the lesion, this method effectively enhances the exposure of the submucosal layer. This technique proves especially beneficial in the gastric angle, where it can be difficult to continue the incision on the antral side. By enhancing visibility and minimizing retraction, dental floss traction plays a crucial role in facilitating safer and more efficient dissection, especially for lesions located in challenging areas.

In conclusion, the application of ESTD particularly when combined with advanced techniques such as dental floss traction and the golden knife, offers a promising alternative to conventional surgical resection for managing large or complex gastric lesions.

## Data Availability

The original contributions presented in the study are included in the article/**Supplementary Material**. Further inquiries can be directed to the corresponding author.

## Supplementary material

Supplementary Figure 1
**(A)** The lesion was entirely resected from both the anal and oral sides. **(B)** This photomicrograph shows the resected specimen with a moderately differentiated adenocarcinoma.

Supplementary Figure 2 An Endoscopic Balloon Dilation Procedure one month After ESTD. **(A)** An endoscopic examination found a scar from a gastric angle ulcer and narrowing in the lower stomach, which extended into the antrum after the ESTD procedure. **(B)** The endoscopic view during the balloon dilation process. **(C)** The endoscopic appearance after Balloon dilation.

Supplementary Figure 3 An Endoscopic Balloon Dilation Procedure three month After ESTD. **(A)** The gastroscopic examination shows the presence of ulcers located in the lower part of the stomach and at the gastric angle, along with a stenosis observed in the pre-pyloric region. **(B)** The endoscopic view during the balloon dilation process. **(C)** The endoscopic appearance after Balloon dilation.

Supplementary Figure 4 it illustrates the findings observed 12 months following ESTD, indicating the absence of stricture **(A)** and recurrence **(B)**.
