# Supplementary figures and images for: Case Report: Endoscopic submucosal tunneling dissection using a golden knife for a giant gastric cancer lesion: a case report and literature review
Source: Front Oncol. 2025 May 30;15:1450446. doi: 10.3389/fonc.2025.1450446 (PMC12162326; doi:10.3389/fonc.2025.1450446)

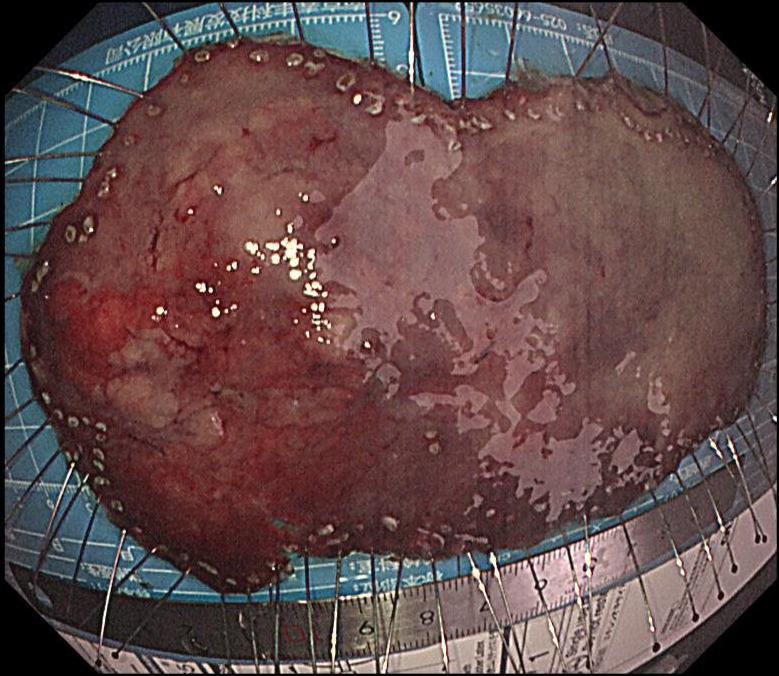

Supplement: Supplementary Figure 1 — (A) The lesion was entirely resected from both the anal and oral sides. (B) This photomicrograph shows the resected specimen with a moderately differentiated adenocarcinoma. [file Image1.jpeg]

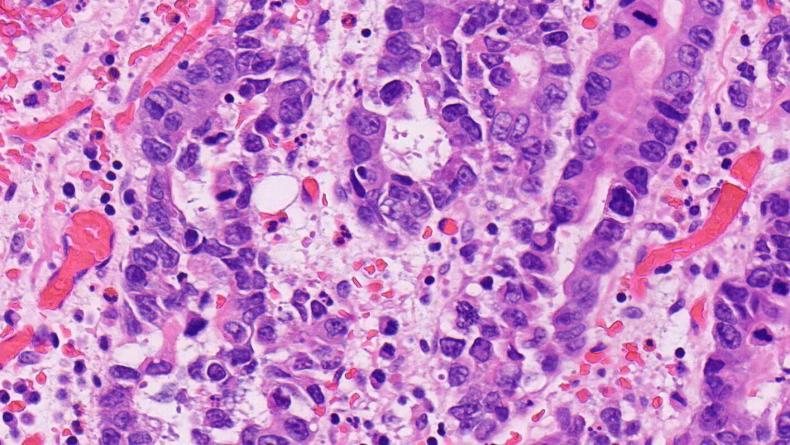

Supplement: Supplementary Figure 2 — An Endoscopic Balloon Dilation Procedure one month After ESTD. (A) An endoscopic examination found a scar from a gastric angle ulcer and narrowing in the lower stomach, which extended into the antrum after the ESTD procedure. (B) The endoscopic view during the balloon dilation process. (C) The endoscopic appearance after Balloon dilation. [file Image2.jpeg]

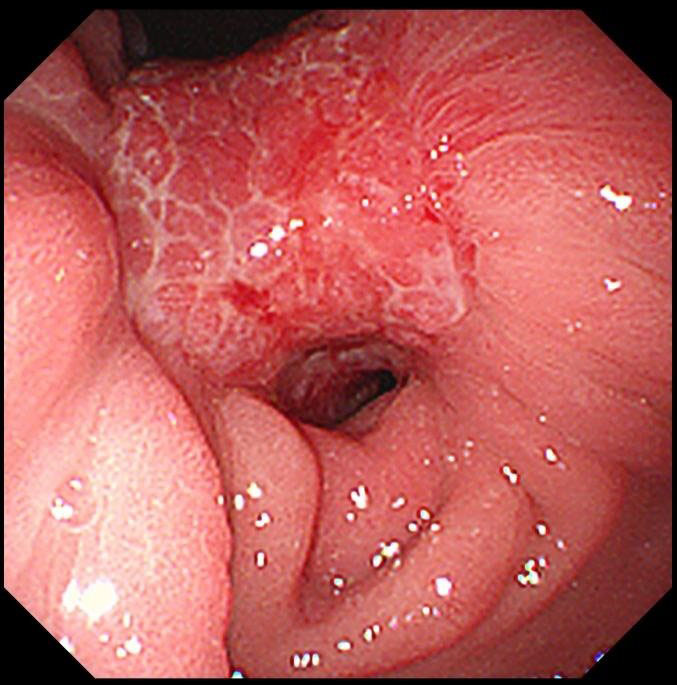

Supplement: Supplementary Figure 3 — An Endoscopic Balloon Dilation Procedure three month After ESTD. (A) The gastroscopic examination shows the presence of ulcers located in the lower part of the stomach and at the gastric angle, along with a stenosis observed in the pre-pyloric region. (B) The endoscopic view during the balloon dilation process. (C) The endoscopic appearance after Balloon dilation. [file Image3.jpeg]

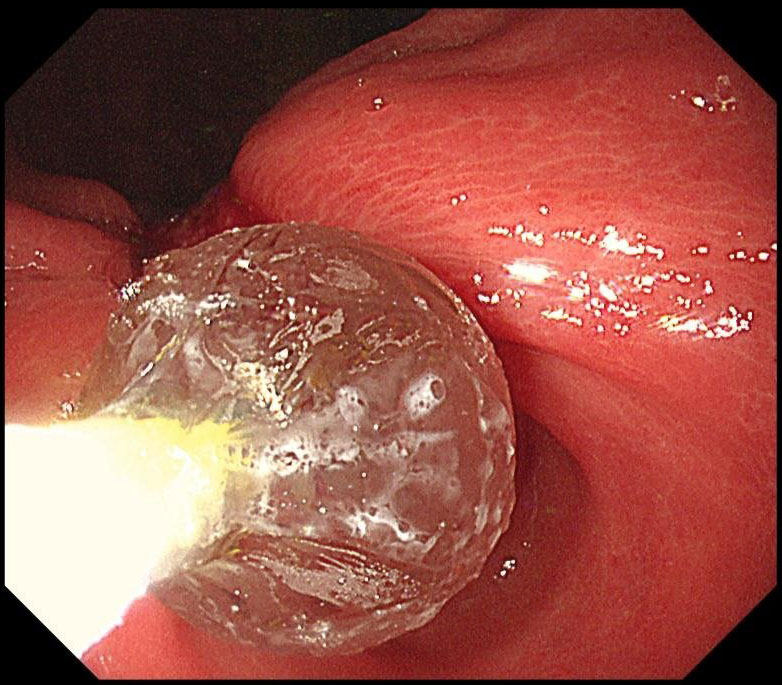

Supplement: Supplementary Figure 4 — it illustrates the findings observed 12 months following ESTD, indicating the absence of stricture (A) and recurrence (B). [file Image4.jpeg]

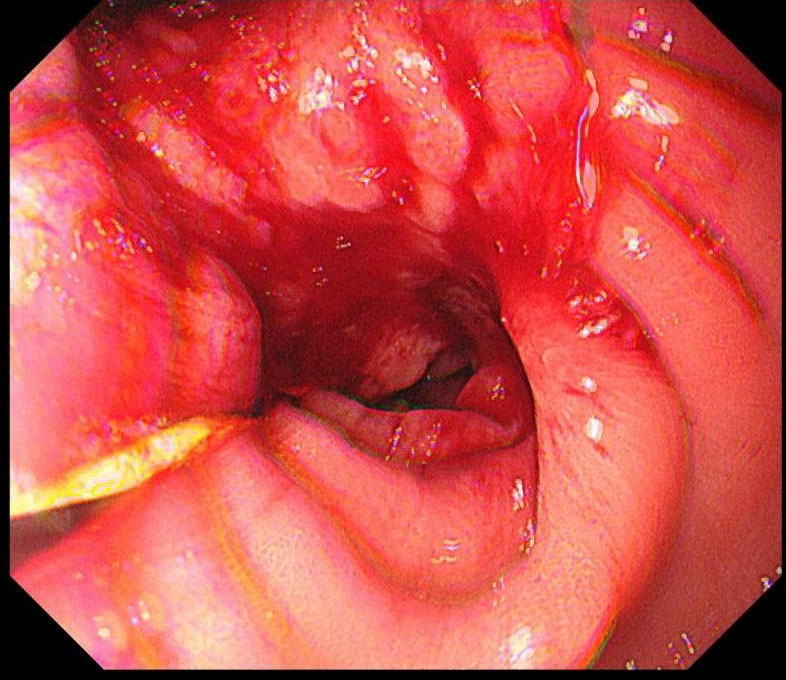

Supplement: Supplementary file 5 [file Image5.jpeg]

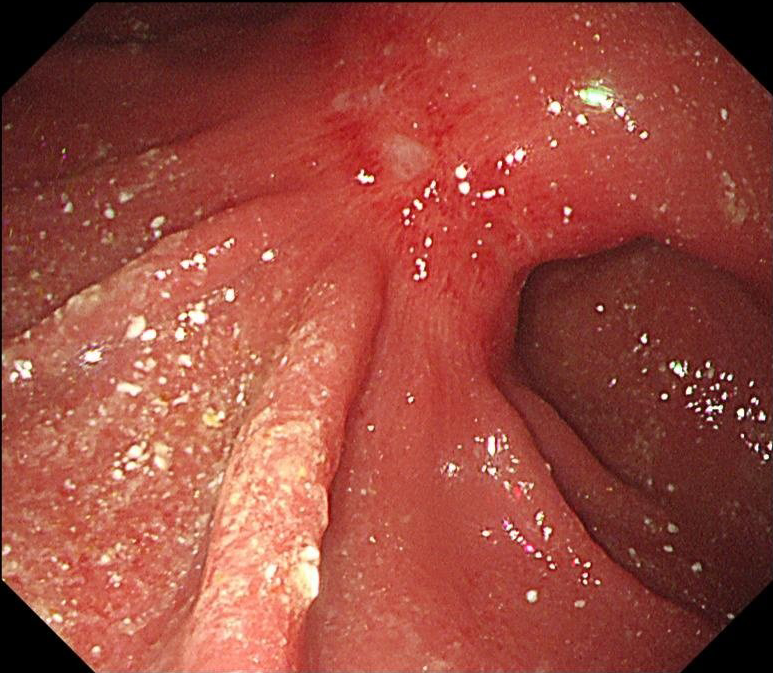

Supplement: Supplementary file 6 [file Image6.tif]

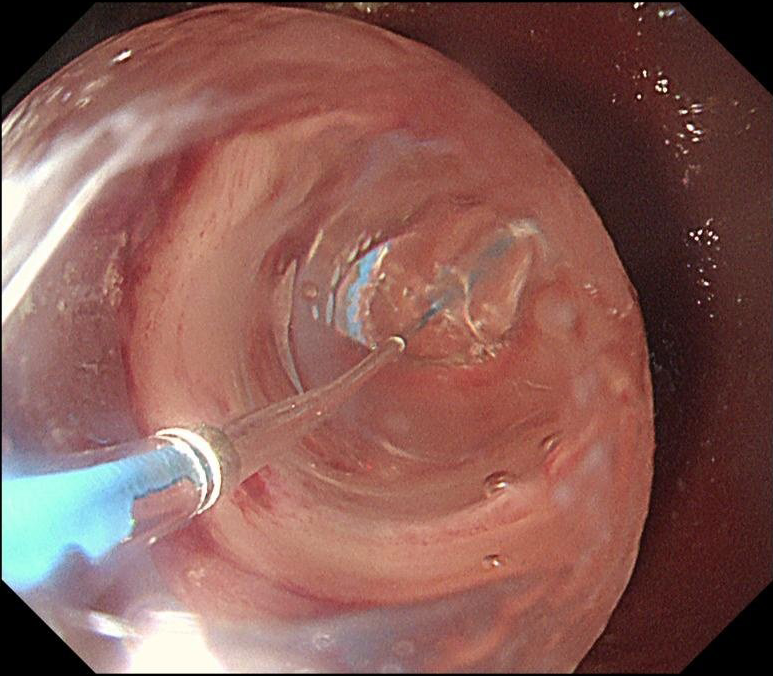

Supplement: Supplementary file 7 [file Image7.tif]

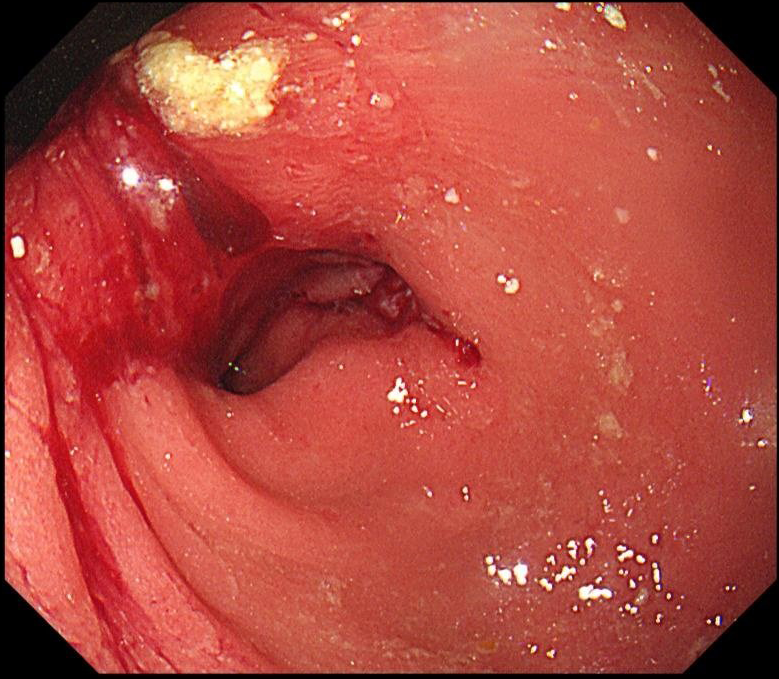

Supplement: Supplementary file 8 [file Image8.tif]

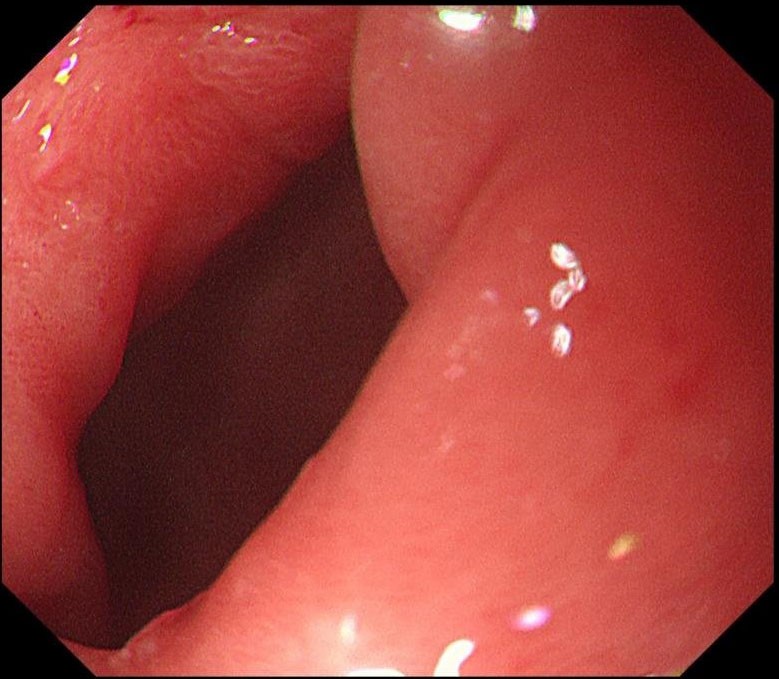

Supplement: Supplementary file 9 [file Image9.jpeg]

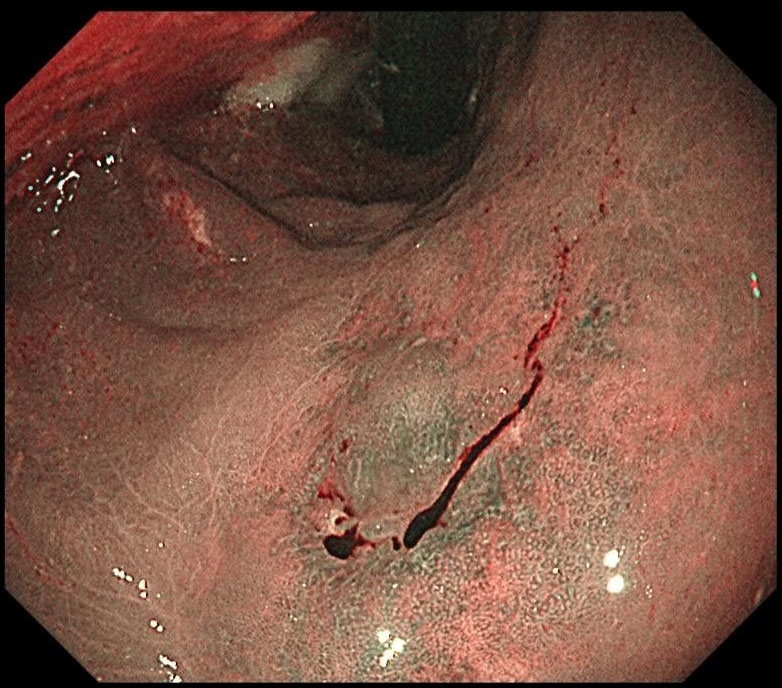

Supplement: Supplementary file 10 [file Image10.jpeg]
